# MicroRNAs and hypospadias: A systematic review

**DOI:** 10.3892/mi.2024.206

**Published:** 2024-11-18

**Authors:** Mahboobeh Amoushahi, Peter Hjorth Jørgensen, Anastasia Buch Kjeldgaard, Eugene Padi, Magdalena Fossum

**Affiliations:** 1 Laboratory of Tissue Engineering, Rigshospitalet, Faculty of Clinical Medicine, University of Copenhagen, 2100 Copenhagen, Denmark; 2 Department of Pediatric Surgery, Center of Cancer and Organ Diseases, Copenhagen University Hospital Rigshospitalet, 2100 Copenhagen, Denmark; 3 Laboratory of Tissue Engineering, Department of Women's and Children's Health, Center of Molecular Medicine, Karolinska Institutet, 17176 Stockholm, Sweden

**Keywords:** microRNA, hypospadias, healing, urology, etiology

## Abstract

The aim of the present study was to summarize what is known about the role of microRNAs (miRNAs/miRs) in relation to hypospadias. Therefore, a systematic review was performed by consulting the electronic databases, MEDLINE (PubMed) and Embase. The search strategy consisted of both MeSH and free text terms, including hypospadias AND miRNA. Only articles written in the English language were included. In the selection process, three authors assessed the studies defined through the search strategy based on titles and abstracts. The authors then analyzed the complete articles and selected studies conforming to the eligibility criteria. Studies that did not align with the adopted criteria were excluded based on the limits set by the initial search strategy: Publication details, study design and participant characteristics. Articles presenting original data on miRNAs associated with hypospadias and including information on miRNA detection and analysis methods were extracted from the selected articles. The included studies presented miRNA extraction from blood samples, preputial or penile tissues, urethral epithelium and/or foreskin fibroblasts. The compiled data focused on the regulatory role of miR-494, miR-145, miR-6756-5p, miR-182, miR-200c, miR-210 and miR-1199-5p in vital cell biological processes, such as wound healing, and cell proliferation, growth and apoptosis. These results proposed that miRNAs may serve as key molecules that merit further investigation; for instance, they may be used as potential biomarkers, diagnostic markers and, eventually, as pharmaceutical agents in hypospadias.

## Introduction

MicroRNAs (miRNAs/miRs) are non-coding RNAs which consist of ~20 nucleotides and have been determined to be critical regulators of eukaryotic gene expression such as in mammals, plants and viruses (1,2). The first miRNA was found >30 years ago in the nematode *Caenorhabditis elegans*, with the detection of the developmental regulator, *lin-4* (3). To date, >2,000 miRNAs have been identified in humans governing one third of the genes in the genome (4). During synthesis, RNA polymerase II transcribes a miRNA gene to produce a long primary miRNA (pri-miRNA), which is cleaved to generate precursor miRNA (pre-miRNA) within the nucleus (5,6). The pre-miRNA is then translocated to the cytoplasm (7-9) where it is cleaved to generate a ~22 nt miRNA duplex (10,11). Of note, one strand of this duplex generates the mature guide miRNA, which binds to an Argonaute protein to become the core of the miRNA-induced silencing complex (miRISC) (12,13). The miRNA region then drives miRISC to complementary sites to govern repression of the target mRNA, while another strand of the mature miRNA duplex, as passenger (opposite) strand, degrades. In mammals, the miRNA target sites, are usually localized in the 3'-untranslated region (3´UTR) of mRNAs, which are known as the sites with the most complementarity to the seed region (14,15). The determination of miRNA-mRNA target interactions is critical for exploring the regulatory role of miRNAs, resulting in the discovery of novel therapies (16).

In recent years, the regulatory roles of miRNAs in various pathologies, ranging from cancer to autoimmune and cardiovascular disease, have attracted major attention (17). By these means, miRNAs possess promising potential for use as biomarkers in diagnostic applications. For example, following the discovery of the regulatory role of miRNAs in cancer, a number of investigations have focused on exploring their potential therapeutic value (18). Currently, the Food and Drug Administration and the European Medicines Agency have accepted a total of 11 RNA-based therapeutics involving small interfering RNA and antisense oligonucleotide. Although miRNAs are not yet approved as therapeutic agents for diseases, some miRNA mimics and anti-miR oligonucleotides are undergoing or completed clinical phase I (NCT01829971, NCT04675996, NCT02369198, NCT04045405) and II (NCT03837457, NCT03373786, NCT02855268 and NCT03601052) trials. For instance, an anti-miR-122 drug, miravirsen, developed for treatment of hepatitis C, has successfully completed clinical phase I (NCT01646489) and II (NCT02508090, NCT02508090, NCT02452814 and NCT02452814) trials (18).

The present systematic review explored what is known regarding miRNAs and hypospadias. Hypospadias is a malformation of the male external genitalia (18.6-80 per 10,000 male births), and it is characterized by a proximal dislocation of the urethral meatus, ventrally split foreskin, and can be combined with penile curvature and transposition of the scrotum (19,20). The moderate to severe cases account for 30%, while the remaining 70% are considered a mild form that is not linked with other urogenital malformations (20).

The etiology of hypospadias is described as complex, meaning that it occurs due to genetic factors in combination with environmental factors. The most well-established risk factor is a low birth weight and growth retardation during gestation (21). In ~10% of cases, hypospadias can be found in an additional family member. Some level of genetic predisposition is established in hypospadias (7% of cases having affected first, second, or third-degree relatives) and hereditary occurrence appears more frequent for distal and middle forms than for posterior varieties (22). The risk of a male sibling also having hypospadias is 9-17% and hypospadias is evenly transmitted through the maternal and paternal side, with an assessed heritability of 57-77% (23). In only 1/3 of hypospadias, an obvious genetic reason is established; however, hypospadias has been described in >200 syndromes, with the most well-known being Wilms' tumor, aniridia, genitourinary malformations, mental retardation (WAGR) and genitourinary malformations and susceptibility to Wilms' tumor (Denys-Drash syndrome) (22,24).

In the present study, it was hypothesized that the genetic predisposition to hypospadias may not be primarily related to mutations in gene-coding regions, but may be caused by silencing intracellular post-transcriptional factors, such as miRNAs. Thus, a systematic review was performed to further elucidate what is known on the subject.

## Data and methods

### Eligibility criteria

The present systematic review was registered in the International Prospective Register of Systematic Reviews (PROSPERO; no. CRD42024502746). The Preferred Reporting Items of Systematic Reviews and Meta-Analyses (PRISMA) guidelines were used for the organization and completion of the study. Only included studies focusing on the association between miRNAs and hypospadias were included, encompassing both human and animal studies. These publications, indexed in formerly two identified databases, were required to have available abstracts, fully accessible online. Moreover, only articles written in the English language where included.

### Search strategy

The present systematic literature review was conducted using the PubMed and Embase databases in September, 2024, including both MeSH and free text terms, with search expression hypospadias AND miRNA (Table I). Furthermore, the reference lists of the identified articles where searched. EndNote 21 (Clarivate) was utilized for the organization and management of reference materials throughout the search process.

### Literature selection

**Figure 1:**
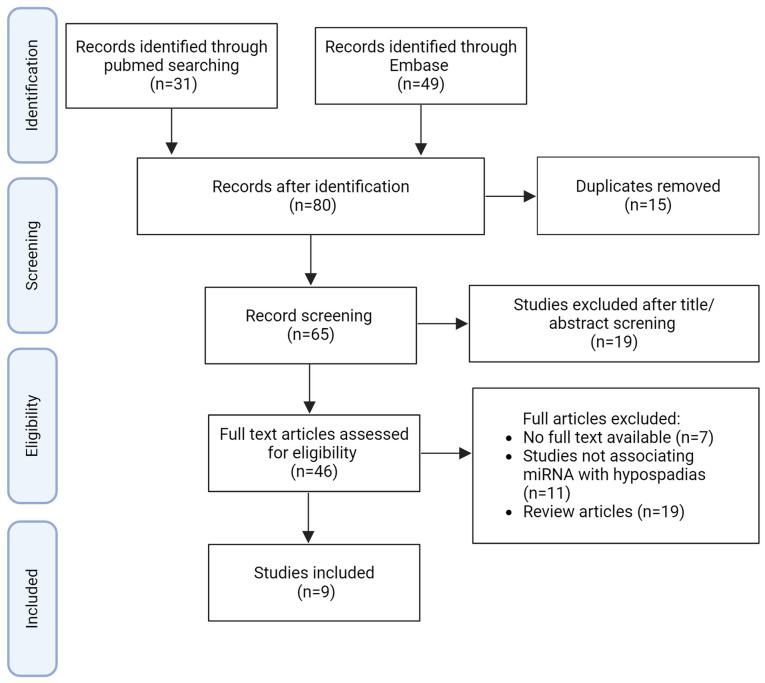
Flow diagram of the search strategy and study selection process for the present systematic review.

The literature search retrieved 65 studies (Fig. 1), whereof 9 studies were selected. The titles and abstracts of all articles identified through the search strategy were independently assessed by three authors involved in the present study (MA, PHJ and MF). These authors also conducted a thorough evaluation of the full articles, applying predetermined eligibility criteria to make their selections. In cases of disagreements the article was discussed, and a consensus was reached.

The extracted data included publication details (first author and year of publication), study design, participant characteristics (mean age, disease type), miRNAs associated with hypospadias, miRNA detection and analysis methods, and the role of miRNAs, as discussed in the original article.

## Results

To the best of our knowledge, no other systemic review was found on the topic. In five studies, the sample species were only human (25-29), in one study, both human and rat samples (30) while in three studies, the sample species were mice (31) and rats (32,33). In total, the compiled studies presented information on miR494, miR-145, miR-6756-5p, miR-182, miR-210, miR-143-3p, miR-566 and miR200-c. The main findings of the selected studies are compiled in Tables II and III.

### Human studies

The range of human sample sizes were from 54 to 1,211 individuals (Table II). The samples were collected from patients with hypospadias of different ages, ranging from 1 to 55 years, with an average age of 5 years. The severity of hypospadias in participants in these studies ranged from mild/moderate to severe. miRNA extraction had been performed from blood samples, urethral epithelial tissues, foreskin fibroblast cells and/or preputial tissues. These human studies indicated the association between miR-6756-5p, miR-182, miR-210, miR-143-3p, miR-556 and miR-145 and hypospadias (25-30). The main analyses applied in these studies were reverse transcription-quantitative polymerase chain reaction (RT-qPCR) and dual luciferase reporter gene assay.

Huang *et al* (25) established a cell line model, using mesenchymal-derived human foreskin fibroblasts (HFF-1), to investigate the role of the hsa-circ-0000417/miR-6756-5p/androgen receptor (AR) axis in PI3K/AKT signaling and in the apoptosis of penile mesenchymal cells. The analysis was conducted using dual luciferase assays, RT-qPCR, flow cytometry and western blot analyses. Their findings indicated that high levels of miR-6756-5p led to the increased proliferation and diminished apoptosis of human foreskin fibroblasts (HFF-1), resulting in the development of hypospadias (25).

Additionally, Deng *et al* (26) examined the impact of miR-182, miR-212, miR-221 and miR-3128 on *Gremlin1* (*GREM1*) expression and the development of hypospadias using luciferase assays. Their study involved 557 patients with hypospadias and 654 healthy controls. They extracted genomic DNA from patient blood samples using TIANamp kits and analyzed the GREM1 rs3743104 SNP via TaqMan genotyping. Bioinformatics tools, TargetScan and MirSNP, were then used to identify potential miRNA binding sites in the GREM1 3'-UTR. They reported that rs3743104 could affect the binding of miR-182 to GREM1, potentially increasing the risk of developing hypospadias (26).

Moreover, Elias *et al* (27) analyzed the expression of miR-210 in plasma from patients with 46,XY investigated for differences in sex development (DSD). Their study involved 36 male controls and 18 patients with 46,XY DSD, with controls divided into the pre-pubertal and post-pubertal subgroups, and patients with DSD categorized based on genitalia features and testicular position. Blood samples were collected, processed and stored for RNA extraction, and miRNA expression was analyzed using RT-qPCR. They demonstrated that miR-210 expression was downregulated in the plasma of patients with 46,XY DSD with hypospadias compared with the control subjects (27).

Peng *et al* (28) demonstrated that miR-143-3p, under the regulatory influence of testosterone, targeted insulin-like growth factor-binding protein 3 (IGFBP-3). They treated HFF-1 cells with testosterone, miR-143-3p mimics, or IGFBP-3 siRNA in various experimental setups to investigate their roles. Transfections were performed using Lipofectamine 2000, with RNA and protein expressions analyzed using RT-qPCR and western blot analysis. Cell viability, migration and luciferase reporter assays were conducted to evaluate cellular responses using specific antibodies and reagents. Their findings indicated that miR-143-3p, by targeting IGFBP-3, inhibited cell growth and reduced AR signaling. This action counteracted the effects of testosterone and may contribute to the development of hypospadias (28).

Furthermore, Chen *et al* (29) conducted a case-control study involving 410 boys with hypospadias and 520 healthy controls between 2009 and 2017. They collected maternal and birth details and excluded participants with a family history of hypospadias. Genotyping of four SNPs (rs12825, rs12458, rs884662 and rs904018) was conducted using TaqMan assays. A dual-luciferase reporter assay revealed that the rs12458 variant could create a binding site for miR-556, potentially influencing *GATA binding protein 4* (*GATA4*) expression and increasing hypospadias risk. They identified miR-556 as a regulator of *GATA4* expression, contributing to the development of hypospadias (29).

In the study by Shang *et al* (30), preputial tissue was collected from pediatric patients following hypospadias repair surgery, and a rat model of hypospadias was established with isolated spermatogonial stem cells. Protein expression related to cell apoptosis and oxidative stress using western blot analysis was analyzed. Additionally, cell apoptosis, proliferation and viability were assessed using flow cytometry, 3-(4,5-dimethylthiazol-2-yl)-2,5-diphenyltetrazolium bromide (MTT) assays and colony formation assays. Their results revealed that miR-145 was highly expressed in hypospadias prepuce. Moreover, their study revealed changes in the expression of miR-377, miR-497, miR-37a and miR-424 in patients with hypospadias compared with healthy controls (30).

### Animal studies

In four identified studies, animal models were applied to investigate the development of hypospadias (30-33). The animal models of hypospadias were created by the daily intragastric administration of Di (2-Ethylhexyl) phthalate (DEHP) or dibutyl phthalate (DBP) to female pregnant mice (one study) or rats (three studies), at different timepoints. The sample size in the mouse study was 34 mice and in the two rat studies used 20 rats in each (Table III). Moreover, in one of the rat studies, the sample size was not specified. In these studies, miRNA was extracted from penile tissue, urethral epithelial tissue, and the genital tubercle. The main applied analyses in the studies were RT-qPCR, dual luciferase reporter gene assay, immunohistochemistry, and western blot analysis. These animal studies demonstrated the association between miR-145, miR-494, miR200-c and miR-1199-5p, and hypospadias. Shang *et al* (30) administered DEHP to female pregnant rats (Table III). In their study, miR-145 expression differed between the hypospadias and control groups.

In the study by Tian *et al* (31), DEHP was administered to female pregnant mice. Following cesarean delivery, male pups with a significantly smaller genital protrusion, urethral orifice at the base of the penis, and bent penis were considered to present a hypospadias phenotype (Table III). Selected pups were euthanized, and the urethral tissues were collected for further analyses, including histological staining and immunohistochemistry to assess tissue morphology and cellular changes. Their results revealed a higher expression of miR-494 in urethral tissue from male mice with hypospadias compared with the control mice (31).

Qian *et al* (32) also administered DEHP to female pregnant rats. They selected newborn rats with hypospadias by measuring the length, curvature and the position of the urethral opening on the penis. Penile tissues were then collected, stored, and analyzed for miR-200c and protein expression using RT-qPCR, immunohistochemistry and western blot analysis techniques. They demonstrated that the downregulation of miR-200c increased the expression of zinc finger E-box binding homeobox 1 (*Zeb1*) in rats, governing the TGF-β/suppressor of mothers against decapentaplegic (Smad)3 pathway and resulting in the formation of hypospadias (32).

Chen *et al* (33) investigated the role of steroid 5 alpha-reductase type 2 (SRD5A2) in cell proliferation, migration, invasion and epithelial-mesenchymal transformation (EMT) in hypospadias. They developed a model of hypospadias by administering DBP daily to healthy pregnant female rats. Primary cells were then isolated from normal and hypospadias rat urethral tissues and cultured in a complete medium at 37˚C with 5% CO_2_. The cells were infected with lentiviral vectors carrying either silenced or overexpressed SRD5A2, selected using puromycin, and further analyzed for gene expression using RT-qPCR and protein expression using western blot analysis. Their findings indicated that SRD5A2 negatively regulated miR-1199-5p and significantly affected key cellular processes in hypospadias, highlighting the critical role of miR-1199-5p in the development of the condition (33).

### Associations between clinical features and miRNAs

To elucidate the role of miRNAs in the clinical manifestation of hypospadias, the present study examined the association between specific miRNAs and clinical features. As regards the association of miRNAs with the severity of hypospadias, miR-494 has been shown to be associated with severe forms of hypospadias (31). This is potentially due to its regulatory effects on the TGF-β1/Smad signaling pathway, which is critical in tissue remodeling and development. The increased expression of miR-494 may be linked to a greater disruption of urethral development, being associated with more severe anatomical presentations (31,34). Conversely, the downregulation of miR-200c expression has been detected in severe hypospadias cases (32). This miRNA is known to regulate EMT processes, which are crucial for proper urethral fusion (35). Its reduced expression suggests a loss of regulation in mesenchymal transition, contributing to severe phenotypes. As regards the association of miRNAs with anatomical characteristics, miR-145 has been implicated in regulating apoptosis and oxidative stress in penile tissues (36,37), suggesting an association with abnormal penile curvature and urethral positioning observed in moderate to severe hypospadias. Its high expression in these tissues indicates that it may contribute to the structural characteristic of the condition.

Moreover, miR-6756-5p has been shown to be associated with alterations in AR signaling, which plays a role in penile and urethral development (25). Higher levels of miR-6756-5p have been linked to the dysregulation of AR expression, indicating a potential link between miRNA levels and specific anatomical anomalies. Furthermore, the interaction of miR-182 with *GREM1* suggests its potential role in hypospadias progression through the bone morphogenic protein (BMP) signaling pathway, which is known to be involved in urethral development (26). Variants in miR-182 may be associated with familial cases or those presenting with additional urogenital anomalies. miR-210, a hypoxia-inducible miRNA, exhibits higher levels in patients with 46,XY DSD and hypospadias, suggesting a link to tissue oxygenation status and potential developmental delays in genital formation (27).

## Discussion

The main purpose of the present systematic review was to explore what was already described regarding the role of miRNAs in hypospadias. The literature was analyzed systematically and a total of nine different miRNAs were determined in the nine selected articles.

In one of the studies, Tian *et al* (31) indicated that miR-494 decreased the expression of Neural precursor cell expressed developmentally downregulated gene 4-like (Nedd4L), resulting in the activation of the TGF-β1/Smad signaling pathway and the promotion of hypospadias. Accordingly, Liu *et al* (38) demonstrated that the higher expression of TGF-β1 promoted the occurrence of hypospadias in DEHP-treated mice. Of note, other studies have demonstrated the essential role of the TGF-β1 signaling pathway in urethral development (38-40). Moreover, the TGF-β signaling pathway and its downstream genes, including activating transcription factor 3, connective tissue growth factor and cysteine-rich angiogenic inducer 61, have been suggested as potential factors in the etiology of hypospadias (38). TGF-β1 governs a vast range of biological processes, such as proliferation, EMT and apoptosis (41-43). EMT is a critical event in urethral embryogenesis (44,45). During this critical biological process, epithelial cells achieve mesenchymal features, such as becoming non-polarized, losing intercellular adhesions and moving throughout the extracellular matrix for tissue generation (46). Previous studies have suggested the transformation of urethral epithelial cells into mesenchymal cells following urethral plate fusion through EMT (44,46). The disruption of the EMT process can cause a failure of the urethral plate fusion, leading to the occurrence of hypospadias (46-48). A recent study determined that miR-1199-5p targets SRD5A2, influencing EMT transformation and promoting the development of hypospadias, highlighting the crucial role of EMT through miR-1199-5p in this condition (33). Moreover, TGF-β1 signaling also plays a critical role in hypospadias development by its regulation of EMT. Notably, recent studies have demonstrated miR-494 as a key regulator of TGF-β1 signaling (49-51).

Previous studies have also demonstrated that miRNAs play a critical role in the regulation of *Nedd4L* expression, which mediates TGF-β1 signaling (52,53). It has been shown that Nedd4L is a critical ubiquitin ligase which selectively targets the activation of Smad2/3 for degradation, resulting in the suppression of TGF-β1 signaling (52). Therefore, miR-494 can indirectly regulate the occurrence of hypospadias. Accordingly, Qian *et al* (32) confirmed the importance of the TGF-β1 signaling pathway in the occurrence of hypospadias through the regulatory role of miR-200c by comparing a rat model of hypospadias induced by DEHP administration with healthy rats. They indicated that there were low levels of miR-200c expression in hypospadias penile tissues, resulting in the high expression of the Zeb1 gene and protein, thereby activating the TGF-b/Smad3 pathway (32). However, both studies included a small sample size, and the degree of hypospadias was not specified in their findings.

miR-145 has been suggested as another potential regulator for the progression of hypospadias in the study by Shang *et al* (30), one of the studies selected for the present systematic review. Previous studies have demonstrated the regulatory role of miR-145 in the expression of pluripotency factors, leading to the reduction of self-renewal and promoting the differentiation of human embryonic cells (54,55). In addition, miR-145 has been demonstrated to affect embryo attachment by reducing the expression of maternal IGF1R. miR-145 has also been shown as a suppressor of phosphorylated extracellular-regulated kinase expression by targeting PAK4, resulting in the reduction of human colorectal cell growth (56). The importance of mitogen-activated protein kinase (MAPK) signaling expands to spermatogenesis and dysgenesis activated by environmental toxicities. This can be ascribed to the compromised blood-testis barrier, leading to reduce semen quality, which could also serve as a contributing factor to hypospadias (57). Of note, MAPK signaling can alter the proper action of downstream SRY-Box transcription actor 9 (SOX9) and the mutation of SOX9 can progress the development of hypospadias (58). SOX9 was found to increase testis development in transgenic mice with XX karyotype (59). Furthermore, the mutation of SOX9 can enhance the progression of campomelic syndrome and sex reversal of XY (60). All the effects of SOX9 on testis development, sex differentiation and hormone secretion suggest a linkage between SOX9 and hypospadias development. Of note, SOX9 has been demonstrated as a target gene of miR-145 for regulating cell activities. Moreover, several studies have shown the accelerating effects of miR-145 on cell apoptosis (61-64). These studies have revealed its inhibitory roles on the proliferation, migration and invasion of the cells through multiple targets, such as Mucin 1, c-Myc, Kirsten rat sarcoma virus, Bax and caspase-3 (61,62).

Cell apoptosis has been demonstrated as a key criterion for normal embryogenesis of male genital tubercles and the anterior urethra. Furthermore, the abnormal alteration of apoptosis in the urethra of mice has been reported in mouse models of hypospadias. Therefore, miR-145 may progress the development of hypospadias through aberrant cell apoptosis. Surprisingly, Shang *et al* (30) suggested the regulatory role of miR-145 in the development of hypospadias was through MAPK signaling and SOX9 expression. They also demonstrated the inhibitory role of miR-145 on nuclear factor erythroid 2-related factor 2-HO-1 signaling and downstream glutathione peroxidase 1 and superoxide dismutase type 1 expression, demonstrating the role of miR-145 as a contributor to hypospadias through the suppression of the antioxidant system. A recent study confirmed the theory of hypospadias development through apoptosis by demonstrating the regulatory role of miR-556 in the *GATA4* expression level (29). *GATA4* has also been shown as a regulator of cell survival (anti-apoptotic) signaling (65). This is supported by findings from the study by Grepin *et al* (66), where the depletion of GATA4 led to apoptosis. These studies demonstrate the involvement of both miR-145 and miR-556 in the development of hypospadias through apoptosis.

miR-6756-5p has been proposed as a potential biomarker involved in the progression of hypospadias. Huang *et al* (25) demonstrated that a high level of miR-6756-5p led to AR suppression, enhanced the activation of PI3K/AKT pathway, the proliferation of HFF-1 cells and impaired apoptosis, potentially contributing to the development of hypospadias. However, the precise functional impact of these molecular events and cellular responses on the progression of hypospadias was not examined in their study. This is a significant limitation as without this information, a direct link between the two cannot be definitively established. Of note, previous studies have indicated that patients with severe hypospadias exhibit a defect in the AR gene (67-70). Specifically, there is evidence suggesting that a crucially reduced level of AR mRNA expression could play a significant role in the development of hypospadias at the midshaft (67). Therefore, miR-6756-5p may play a pivotal role as a post-transcriptional regulator of AR in genital mesodermal cells, which could potentially contribute to hypospadias driven by AR loss in humans. Peng *et al* (28) treated HFF-1 cells with testosterone and revealed that miR-143-3p, by targeting IGFBP-3, impede cell growth and decrease AR signaling, thereby counteracting testosterone, leading to the progression of hypospadias. Therefore, miR-143-3p can be another potential regulator of AR signaling, resulting in the regulation of hypospadias development. Notably, it should be considered that their study did not explore the detailed mechanism or the direct connection between AR signaling and the progression of hypospadias. Furthermore, all their findings were at the cellular level and require validation in human and animal samples. miR-143-3p has also been shown to modulate specific target genes associated with cancer, cardiovascular disease and other health conditions, influencing the progression of these diseases in numerous studies (71-74). Furthermore, studies have indicated that IGFBP-3 plays a role in influencing the progression of various diseases (75-77). This is achieved by inhibiting receptor binding and impeding their anti-apoptotic functions. Additionally, IGFBP-3 has been reported to hinder angiogenesis and impact apoptosis (78-80).

Deng *et al* (26) also discovered that miR-182 can target the seeding region encompassing rs3743104 within the 3'-UTR of GREM1, which in turn inhibits GREM1 transcription. miR-182 has previously been documented as a biomarker for kidney injury and bladder cancer (81,82). It functions as a regulatory miRNA by binding to the 3'-UTR of target genes, influencing the progression of diseases (81). GREM1 is recognized as part of the bone morphogenic protein antagonist family (BMP) (82), and has been linked to susceptibility to hypospadias. Although these findings did not demonstrate a direct link between miR-182 and the progression of hypospadias, miR-182 could potentially play a key role in advancing the development of hypospadias through the BMP signaling pathway.

miR-210 may serve as another regulatory key in the onset of hypospadias. Elias *et al* (27) measured the plasma level of miR-210 in patients with 46,XY DSD with atypical genitalia, demonstrating a higher expression of miR-210 in hypospadias compared with the control group. It has been shown that miR-210 directly targets IGF2 through *in vitro* experiments conducted in NT2 cells. Consequently, these findings suggest a potential association between miR-210 and Insulin/IGF signaling. However, it should be considered that their study involved a small group of patients. Prior research has demonstrated that miR-210 expression is elevated in the testes of infertile men with maturation arrest (83,84). Moreover, miR-210 exhibits a ubiquitous expression across a diverse range of cells, playing physiological roles, such as suppressing cell proliferation, mitochondrial respiration and DNA repair, and affecting vascular biology and angiogenesis (85-87). Recognized as a major hypoxia-inducible miRNA, its relevance is noteworthy in conditions, such as placental hypertension and preeclampsia, which can induce fetal hypoxia (88). A previous study demonstrated that insulin/IGF and hypoxia signaling act in concert in *Caenorhabditis elegans* (89). Therefore, miR-210 can promote the development of hypospadias through insulin/IGF1 and hypoxia signaling.

Surprisingly, several miRNAs in the reviewed articles, including miR-494, miR-145, miR-6756-5p, miR-182, miR-210 and miR-200c, were found to be associated with key clinical features of hypospadias, such as disease severity and specific anatomical abnormalities. miR-494 was found to be associated with more severe forms of the condition, potentially due to its impact on TGF-β1/Smad signaling pathways. Moreover, the downregulation of miR-200c was linked to impaired EMT processes in urethral development. To investigate the role of miRNAs in the clinical presentation of hypospadias, the association between specific miRNAs and key clinical characteristics was analyzed. The analysis suggested that specific miRNAs could serve as biomarkers for predicting the severity and anatomical characteristics of hypospadias. Further studies with larger sample sizes are required however, to validate these findings and explore the direct mechanistic roles of these miRNAs in hypospadias.

**Figure 2:**
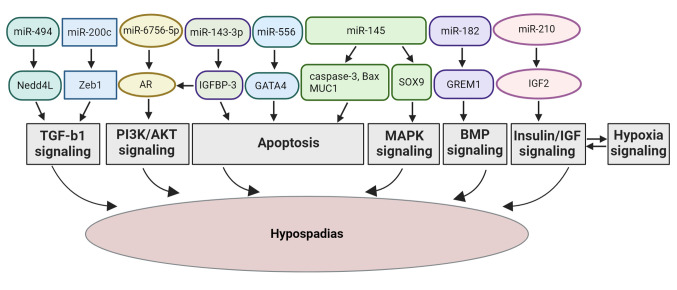
Schematic diagram presenting the mechanisms through which the regulatory roles of miR-494, miR-200c, miR-145, miR-6756-5p, miR-143-3p, miR-182, miR-210 and miR-556 can lead to hypospadias through the TGF-β, MAPK, PI3K/AKT, AR, BMP, insulin/IGF, hypoxia signaling pathways, and apoptosis, respectively. MiR, microRNA; Nedd4L, neural precursor cell expressed developmentally downregulated 4-like; TGF-β1, transforming growth factor-β1; Smad, suppressor of mothers against decapentaplegic; SOX9, sex-determining region Y box 9; MAPK, mitogen-activated protein kinase; Zeb1, zinc finger E-box binding homeobox 1; IGFBP-3, insulin-like growth factor binding protein; AR, androgen receptor; PI3K/AKT, phosphoinositide 3-kinase/protein kinase B; GREM1, Gremlin-1; GATA4, GATA binding protein 4; IGF2, insulin-like growth factor 2.

The present systematic review provides a comprehensive analysis of the role of specific miRNAs in the development of hypospadias, integrating evidence from both human and animal studies. By highlighting miRNAs, such as miR-494, miR-145, miR-6756-5p, miR-182, miR-210, miR-143-3p, miR-556 and miR-200c, the present systematic review identifies key molecular pathways, including TGF-β, MAPK, PI3K/AKT, BMP and AR signaling (Fig. 2), that are crucial in regulating cellular processes, such as proliferation, apoptosis and differentiation resulting in hypospadias progression. The findings suggest that miRNAs do not merely function as biomarkers, but actively participate in disease progression, linking genetic predispositions with environmental influences. This integrative approach provides valuable insight into the molecular mechanisms underlying hypospadias and underscores the potential of miRNAs as therapeutic targets, setting the stage for future translational research aimed at developing novel diagnostic and treatment strategies.

However, the included studies faced significant limitations, including small sample sizes, a lack of stratified analyses, the absence of specific treatment groups and findings limited to cellular level, which restricted the understanding of the molecular mechanisms and the direct links between miRNAs and hypospadias progression. Further investigations are required to validate these findings in human and animal samples and to explore the detailed roles of miRNAs in the development of hypospadias. A limitation of the present systematic review is that the literature search was conducted using only two databases, which may have restricted the comprehensiveness of the review and may have thus potentially excluded relevant studies. Several potential biases in the included studies have been highlighted throughout the present systematic review. Small sample sizes reduce the statistical power and limit generalizability, while selection bias arises from focusing on specific populations, such as pediatric patients and animal models. Language bias from including only studies in the English language and publication bias favoring significant results may skew the findings. Variability in experimental methods and the lack of stratified analyses further complicate comparisons across studies. The majority of the studies were observational, lacked validation in human samples, and often relied on single time point measurements, which may miss dynamic changes. Additionally, confounding factors, such as environmental exposures and genetic variability were not consistently controlled, limiting the ability to establish the link between miRNAs and hypospadias.

In conclusion, the present systematic review demonstrated the link between miR-494, miR-145, miR-6756-5p, miR-182, miR-200c, miR-210 and miR-1199-5p, and the occurrence of hypospadias. The majority of the included articles focused on etiological factors. Notably, miR-494, miR-200c, miR-145, miR-6756-5p, miR-182 and miR-210 could promote the development of hypospadias through different signaling pathways, such as TGF-β, MAPK, PI3K/AKT, BMP, insulin/IGF and hypoxia. These pathways are also involved in tissue regeneration and wound healing. Therefore, the present systematic review suggests that miRNAs may serve as potential biomarkers related to hypospadias and may play a role in potentiating wound healing following surgery. Further investigations are required however, to shed light onto the regulatory role of miRNAs in the development of hypospadias, which can provide a novel direction for the understanding of the etiology of hypospadias and improving treatment modalities.

## Figures and Tables

**Table I:** Descriptors used in the search strategy.

Topic	Descriptors
MicroRNAs	‘MicroRNAs’[MeSH] OR microRNA* OR miRNA* OR Micro RNA OR Micro RNAs OR MiR OR ‘Primary MicroRNA’ OR ‘Primary miRNA^*^’ OR Pri-miRNA^*^ OR Pri miRNA^*^ OR ‘Temporal RNA, small’ OR ‘RNA, small temporal’ OR stRNA* OR ‘Small temporal RNA’ OR Pre-miRNA* OR Pre miRNA* OR MicroRNA* OR miRNA^*^ OR ‘Micro RNA’ OR ‘Micro RNAs’ OR MiR OR ‘Primary MicroRNA’ OR ‘Primary miRNA*’ OR Pri-miRNA* OR Pri miRNA* OR ‘Temporal RNA, small’ OR ‘RNA, small temporal’ OR stRNA^*^ OR ‘Small temporal RNA’ OR Pre-miRNA* OR Pre miRNA*
Hypospadias	‘Hypospadias’[MeSH] OR Hypospadia* OR ‘Urethral development’ OR ‘Urogenital malformation*’ OR ‘Disorder of sexual development’ OR ‘development of urethra’ OR ‘Urethra development’ OR ‘Urethra malformation*’ OR DSD OR ‘Genital malformation*’ OR Hypospadia* OR ‘Urethral development’ OR ‘Urogenital malformation*’ OR ‘Disorder of sexual development’ OR ‘development of urethra’ OR ‘Urethra development’ OR ‘Urethra malformation*’ OR DSD OR ‘Genital malformation*’

As regards the result of the MeSH search, in the list of MeSH headings assigned to a citation that appear in the PubMed record, a MeSH Major Topic is denoted by an asterisk (*) on the MeSH term or MeSH/Subheading combination. MeSH, Medical subject headings.

**Table II:** Summary of literature search findings in humans.

Authors, year of publication	Study design	Sample (size/cell line number)	Age in human samples	Type of hypospadias	Type of experiments	Key results/comments	(Refs.)
Peng *et al*, 2023	Experimental/randomized parallel group	Commercial HFF-1 cell line (CBP60935)	-	-	RT-qPCR, luciferase assay and Transwell assay to investigate the involvement of testosterone in hypospadias through miR-143-3p/IGFBP-3 axis.	miR-143-3p can bind to IGFBP-3 by regulatory role of testosterone, activating HFF-1 cells growth and AR signaling, resulting in hypospadias occurrence.	(28)
Elias *et al*, 2022	Case-control study	Blood samples of patients with 46, XY DSD (n=18) and healthy (n=36) individuals	1-55 years	Second- or third-degree hypospadias	RT-qPCR to study the relationship between miR-210 plasma level and atypical genitalia at birth.	Plasma levels of miR-210 expression may have a positive association with presence of atypical genitalia in the patients with 46, XY DSD.	(27)
Deng *et al*, 2021	Case-control study	Venous blood samples of hypospadias (n=557) and healthy (n=654) individuals	*Unknown*	Mild/moderate: coronal or glanular or shaft penis hypospadias; Severe: penoscrotal, scrotal and perineal hypospadias	Bioinformatic analysis and luciferase assay to study the role of miR-182 and GREM1 in development of hypospadias.	rs3743104 can govern GREM1 expression by miR-182 leading to impact development of hypospadias.	(26)
Chen *et al*, 2021	Case-control study	Penile skin tissue, and 293 cell line	*Unknown*	Anterior: glandular, coronal, and subcoronal, middle: penile, and posterior: penoscrotal, scrotal, and perineal	SNP Selection and genotyping, and luciferase assay to study the effect of GATA4 polymorphisms on hypospadias incidence.	miR-566 can be a regulator of GATA4 expression, involving in development of hypospadias.	(29)
Huang *et al*, 2023	Experimental/randomized parallel group	Commercial HFF-1 cell line (ATCC number: SCRC-1041)	-	-	RT-qPCR, luciferase assay, in situ hybridization and flow cytometry to explore the role of hsa-circ-000417/miR-6756-5p/AR axis in penile mesenchymal cell proliferation and apoptosis.	hsa_circ_0000417/miR-6756-5p/AR/p-AKT axis may play a regulatory role in the development of hypospadias by reducing apoptosis of penile mesenchymal cells.	(25)
Shang *et al*, 2019	Experimental/randomized parallel group	Hypospadias tissues (n=208), preputial tissues (n=241)	1-4 years	Mild/moderate: Urethral opening between distal and middle penis. Severe: Urethral opening between middle and radix penis, scrotum, or perineum	Study of miR-145 role in hypospadias development in human and rat hypospadias model following by microarray, Luciferase assay, western blotting, and flow cytometry.	miR-145 may have regulatory role in the development of hypospadias through SOX9 and MAPK signaling pathway.	(30)

RT-qPCR, reverse transcription-quantitative polymerase chain reaction; IGFBP-3, insulin-like growth factor binding protein; HFF-1, human foreskin fibroblast c*ell l*ine; *SOX9, s*ex-determining region Y box 9; MAPK, mitogen-activated protein kinase; AR, androgen receptor; AKT, protein kinase B; GREM1, Gremlin-1; DSD, disorder of sex development.

**Table III:** Summary of literature search findings in animals.

Authors, year of publication	Study design	Species (sample size)	Model construction	Type of hypospadias	Type of experiments	Key results/comments	(Refs.)
Chen *et al*., 2024	Experimental/randomized parallel group	Rat (n=unknown, primary cells from normal and hypospadias urethral tissues)	Intragastric administration of DBP in female pregnant rats at GD 14-18	Unknown	RT-qPCR, luciferase assay, and western blotting of primary cells isolated from normal and hypospadias rat urethral tissues to determine the role of SRD5A2 in the development of hypospadias through miR-1199-5p.	SRD5A2 downregulated miR-1199-5p and significantly influences key cellular processes in hypospadias, underscoring the pivotal role of miR-1199-5p in the development of this condition.	(33)
Tian *et al*., 2020	Experimental/Randomized parallel group	Mouse (n=17 in each group)	Intragastric administration of DEHP in female pregnant mice every day from GD12 to GD19	i) A significantly smaller genital protrusion, a clear fissure in the abdomen, not prominent testis, female appearance; ii) the urethral orifice close to side of the penis, and bent penis	Study of miR-494 role in mouse model of hypospadias by RT-qPCR, luciferase assay, western blotting, and flow cytometry.	miR-494 can progress hypospadias through binding to Nedd4L and activation of TGF-β1/Smad signaling pathway.	(31)
Shang *et al*., 2019	Experimental/randomized parallel group	Rat (n=20 in each group)	Intragastric administration of DEHP in female pregnant rats at GD 14-18	Mild/moderate: Urethral opening between distal and middle penis; Severe: Urethral opening between middle and radix penis, scrotum, or perineum	Study of miR-145 role in hypospadias development in human and rat hypospadias model following by microarray, luciferase assay, western blotting, and flow cytometry	miR-145 may have regulatory role in development of hypospadias through SOX9 and MAPK signaling pathway.	(30)
Qian *et al*., 2016	Experimental/randomized parallel group	Rat (n=20 in each group)	Intragastric administration of DEHP in female pregnant mice every day from GD12 to GD19	Hypospadias of rats were diagnosed based on length, curvature, and urethral opening position of penis	RT-qPCR, luciferase assay, immunohistochemistry and western blotting of rat hypospadias model to explore the effect of miR-200c in development of hypospadias.	MiR-200c can promote hypospadias through TGF-β1/Smad3 signaling pathway by increasing the expression of Zeb1.	(32)

RT-qPCR, reverse transcription-quantitative polymerase chain reaction; Nedd4L, neural precursor cell expressed developmentally down-regulated 4-like; TGF-β1: transforming growth factor-β1; Smad: suppressor of mothers against decapentaplegic; *SOX9*, sex-determining region Y box 9; MAPK, mitogen-activated protein kinase; Zeb1, zinc finger E-box binding homeobox 1; DEHP, di(2-ethylhexyl) phthalate; DBP: dibutyl phthalate; GD, gestation day; SRD5A2, steroid 5 alpha-reductase type 2.

## Data Availability

The datasets used and/or analyzed during the current study are available from the corresponding author on reasonable request.
